# Vitamin and Mineral Supplementation Practices in Preterm Infants: A Survey of Australian and New Zealand Neonatal Intensive and Special Care Units

**DOI:** 10.3390/nu12010051

**Published:** 2019-12-23

**Authors:** Colleen Oliver, Caitlin Watson, Elesa Crowley, Melissa Gilroy, Denise Page, Katrina Weber, Deanna Messina, Barbara Cormack

**Affiliations:** 1 Department of Dietetics and Nutrition, The Royal Women’s Hospital, Melbourne, VIC 3052, Australia; 2 Department of Dietetics and Nutrition, Monash Children’s Hospital, Melbourne, VIC 3168, Australia; Caitlin.watson@monashhealth.org; 3 Department of Rural Health, Faculty of Health and Medicine, The University of Newcastle, Tamworth, NSW 2340, Australia; elesa.crowley@newcastle.edu.au; 4 Department of Dietetics and Nutrition, Tamworth Rural Referral Hospital, Tamworth, NSW 2340, Australia; 5 Department of Dietetics and Food Services, Mater Group, South Brisbane, QLD 4101, Australia; Melissa.gilroy@mater.org.au (M.G.); denise.page@mater.org.au (D.P.); 6 Department of Dietetics and Nutrition, Fiona Stanley Hospital, Perth, WA 6150, Australia; katrina.weber@health.wa.gov.au (K.W.); Deanna.messina@health.wa.gov.au (D.M.); 7 Department of Dietetics and Nutrition, Starship Child Health, Auckland City Hospital, Auckland 1023, New Zealand; bcormack@adhb.govt.nz; 8 Liggins Institute, University of Auckland, Auckland 1142, New Zealand

**Keywords:** neonatal, preterm, vitamin, mineral, supplementations

## Abstract

Preterm infants are at increased risk of micronutrient deficiencies as a result of low body stores, maternal deficiencies, and inadequate supplementations. The aim of this survey was to investigate current vitamin and mineral supplementation practices and compare these with published recommendations and available evidence on dosages and long-term outcomes of supplementations in preterm infants. In 2018, a two-part electronic survey was emailed to 50 Australasian Neonatal Dietitians Network (ANDiN) member and nonmember dietitians working in neonatal units in Australia and New Zealand. For inpatients, all units prescribed between 400 and 500 IU/day vitamin D, compared to a recommended intake range of 400–1000 IU/day. Two units prescribed 900–1000 IU/day at discharge. For iron, 83% of respondents prescribed within the recommended intake range of 2–3 mg/kg/day for inpatients. Up to 10% of units prescribed 6 mg/kg/day for inpatients and at discharge. More than one-third of units reported routine supplementations of other micronutrients, including calcium, phosphate, vitamin E, and folic acid. There was significant variation between neonatal units in vitamin and mineral supplementation practices, which may contribute to certain micronutrient intakes above or below recommended ranges for gestational ages or birth weights. The variations in practice are in part due to differences in recommended vitamin and mineral intakes between expert groups and a lack of evidence supporting the recommendations for supplementations.

## 1. Introduction

Preterm infants are at high risk of micronutrient deficiencies due to maternal deficiencies, low body stores at birth, and low nutritional intakes, such that most preterm infants require vitamin supplementations soon after birth [1]. With the exception of vitamin D, the intake of most micronutrients is met by fortified breast milk and preterm infant formula feeds at enteral feed volumes greater than 150 ml/kg/day. Whilst similar amounts of vitamins and minerals are generally provided by most commercial preterm nutritional products, individual requirements may vary significantly depending on gestational ages, stores at birth, and clinical factors.

Several international consensus recommendations exist for daily micronutrient intakes for fully enterally fed, stable-growing preterm infants with birth weights up to 1500 g (very low birth weights; VLBWs, Table 1). The most recent of these, Koletzko et al. 2014, is based on current knowledge and expert group discussions [1]. Similar published recommendations on nutrient intakes are not available for low birth weight (LBW; <1.5–<2.5 kg) babies. 

Estimates of requirements for calcium and phosphorus in preterm infants are based on studies of intrauterine mineral accretion rates and calcium absorption and retention. Most studies in preterm infants have based vitamin D sufficiencies on the Institute of Medicine’s definition of a 25-hydroxyvitamin D level (>50 nmol/L), which is based on biomarkers from adult studies; however, no definition of vitamin D deficiencies based on biological functions is available for preterm infants [4]. In addition to having a negative impact on bone health, low neonatal vitamin D levels have been associated with greater risks of respiratory distress syndrome [5], bronchopulmonary dysplasia (BPD) [6], and sepsis [7]. 

Iron deficiencies during infancy in healthy term infants are associated with irreversible, long-term neurodevelopmental impairments [8,9]. Early iron supplementations in VLBW infants have been shown to be safe and reduce the risk of iron deficiencies [10,11]; however, it is unclear whether iron supplementations in preterm infants have long-term benefits for growth and neurodevelopmental outcomes.

The evidence base is limited for quantifying ideal intakes of other micronutrients and long-term outcomes. Fat-soluble vitamins A and E act as antioxidants, and pharmacological doses may confer benefits for high-risk infants in the prevention of BPD, retinopathy of prematurity (ROP), and necrotising enterocolitis (NEC) [12]. A lack of data exists on the potential toxicity and long-term effects of high doses of fat-soluble vitamins.

The lack of evidence for optimal doses and long-term safety has resulted in significant practice variations across neonatal units, and subsequently, micronutrient intakes above or below recommended ranges. In addition, the continuation of historical practices and a lack of clinician knowledge about the nutritional compositions of preterm nutritional products may result in unnecessary supplementations. The aim of this survey was to investigate current vitamin and mineral supplementations practices in Australian and New Zealand (NZ) neonatal intensive care units (NICUs) and special care nurseries (SCNs) and compare these with consensus recommendations and published evidence on dosages and outcomes of supplementations in preterm infants.

## 2. Materials and Methods 

As part of a wider study of neonatal nutrition practices and dietitian resourcing, a link to a two-part electronic pretested survey (Survey Monkey, SVMK Inc., San Mateo, CA, USA) consisting of multiple-choice questions was emailed on 16 July, 2018 to 50 Australasian Neonatal Dietitians Network (ANDiN) members working in a NICU or SCN in Australia or New Zealand. Non-ANDiN member neonatal dietitians practising in Australia or New Zealand were also invited to participate. Non-responders were sent a reminder email after four weeks and again just prior to the survey closing. If no response was received from a level 3 NICU site listed in the Australian and New Zealand Neonatal Network (ANZNN) directory, an email was sent to a neonatologist at the site to provide contact details for the neonatal dietitian or confirm that they did not have a dietitian. The survey closed on 10 October, 2018. Responses were stored on a password-protected online site.

Survey questions for Part 2 of the survey can be found in Supplementary Materials. The Checklist for Reporting Results of Internet E-Surveys (CHERRIES) statement guidelines were followed for reporting survey results. Descriptive statistics were used to examine the distribution of survey responses. Percentages were calculated for categorical variables.

## 3. Results

Part two of the survey referred to site practices. Responses were received from 35 sites, 10 (29%) from NZ and 25 (71%) from Australian sites. Twenty-three (79%) responses were received from the 29 ANZNN level 3 NICU sites. One response covered two NICU sites. We confirmed one NZ and three Australian level 3 NICUs had no neonatal dietitians. There were nine (26%) site responses from ANDiN members currently working in 34 level 2 SCNs. Written guidelines for inpatient vitamin and mineral supplementations were reported to exist in 86% of units. 

### 3.1. Vitamin D Supplementation

**Figure 1:**
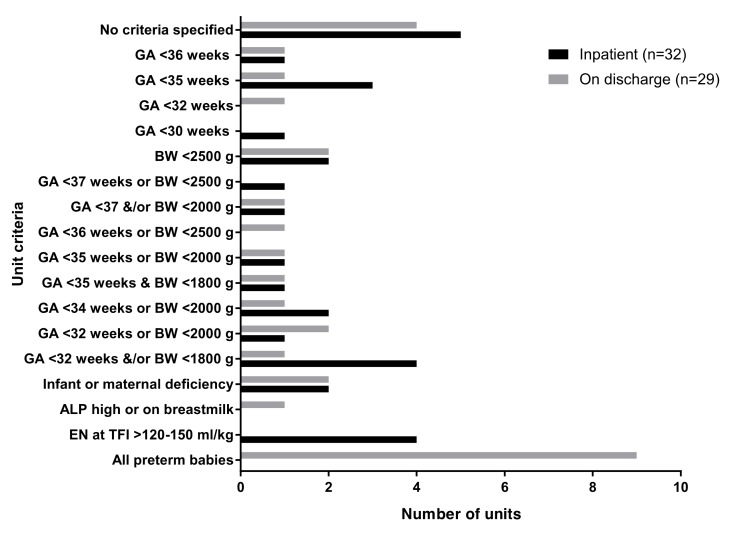
Supplementation criteria for vitamin D. GA = gestational ages; BW = birth weights; ALP = alkaline phosphatase; EN = enteral nutrition; and TFI = total fluid intakes.

Of the 32 responses received, all neonatal and special care units routinely prescribed vitamin D for inpatients. One unit did not prescribe ongoing vitamin D supplements at discharge. Supplementation criteria were based on a wide range of gestational ages and birth weights (Figure 1). 

**Figure 2:**
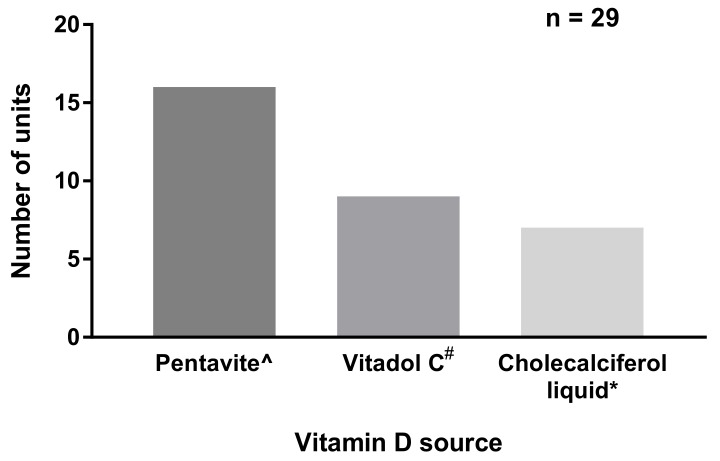
Source of vitamin D prescribed. ^ = multivitamin preparations containing vitamins A, D, C, B^1^, B^2^, B^3^, and B^6^; # = multivitamin preparations containing vitamins A, D, and C; and * = vitamin D_3_ liquid.

**Figure 3:**
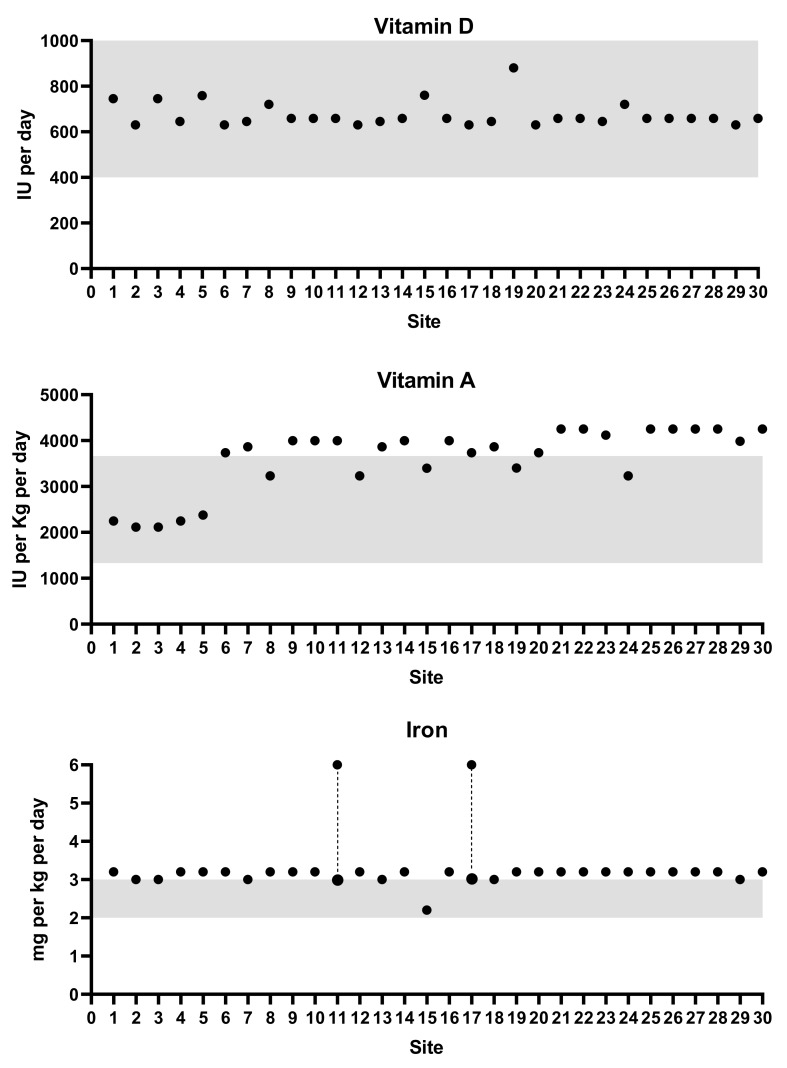
Total intakes^∆^ compared to recommended nutrient intakes for vitamin D, vitamin A, and iron. ^∆^ = total nutrient intakes calculated for each site based on a 1 kg infant using the reported target feed volume, brand of breastmilk fortifier, and type of vitamin or mineral supplement used. Shaded areas represent the recommended intake range for each nutrient. Dashed lines indicate units that prescribed a range of iron doses.

Vitamin D was supplemented in the form of a multivitamin in half of the Australian sites and in 90% of the NZ sites (Figure 2). Seven (22%) Australian sites prescribed a vitamin D_3_ liquid (cholecalciferol). All Australian and NZ units prescribed between 400 and 500 IU vitamin D intakes for inpatients, resulting in total vitamin D intakes from feeds and supplements ranging between 630 and 880 IU per day (Figure 3; based on a 1 kg infant). The same doses were prescribed at discharges in all but one NZ and one Australian unit, both of which were prescribed 900–1000 IU intakes per day.

Most units surveyed (73%) ceased vitamin D supplements between 6–12 months corrected age (CA) for prematurity. The remaining units ceased supplements within the first three months CA (7%) upon normalisation of biochemical markers for metabolic bone disease (3%) or at the discretion of the GPs or paediatricians managing care post-discharge (17%).

### 3.2. Iron Supplementation

**Figure 4:**
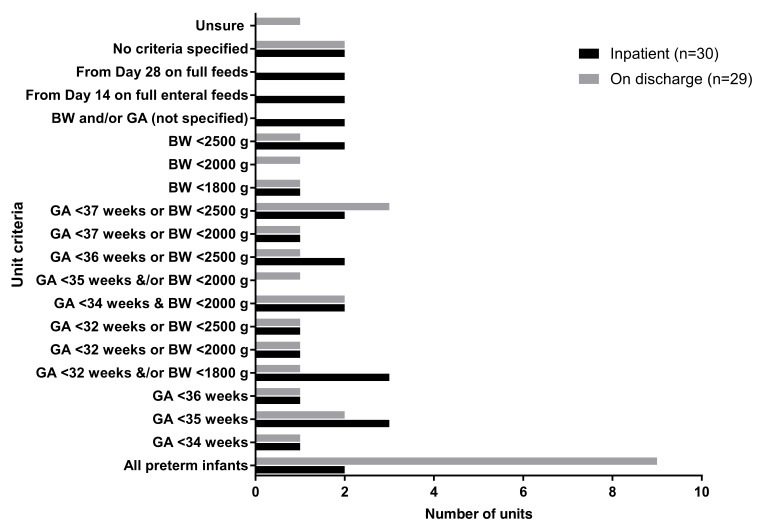
Supplementation criteria for iron. GA = gestational ages and BW = birth weights.

Iron was prescribed for inpatients in the form of ferrous sulphate or an iron-containing human milk fortifier in the 31 units that responded. In the majority, supplementation criteria were based on gestational ages or birth weights (Figure 4). Most units did not commence ferrous sulphate until iron-containing breast milk fortifiers or preterm formulas were discontinued. Gestational ages less than 37 weeks was the main criteria for supplementations at discharge. 

More than 80% of units prescribed a ferrous dose between 1.8 and 3 mg/kg/d for both inpatients and outpatients (Table 2), resulting in total iron intakes of around 3 mg/kg/d for a 1 kg infant, the upper range of recommended intakes (Figure 3). Three (10%) units provided up to twice the recommended intakes of iron at the time of discharge (Table 2). Supplements were stopped at six months CA or under in 40% of units and between 6–12 months corrected age in 50% of units. The remaining units ceased supplements if an infant was fully formula-fed or at the discretion of the GP or paediatrician.

### 3.3. Other Vitamins and Minerals

Of the 27 units that responded, 63% did not routinely prescribe any other vitamins or mineral supplements for inpatients. Of the 10 units that did prescribe, almost 50% prescribed calcium and/or phosphate, 40% prescribed vitamin E, and 30% prescribed folic acid. 

In units prescribing a multivitamin for vitamin D supplementation, total vitamin A intakes (from oral supplements and fortified milk feeds) exceeded the upper recommended intake for vitamin A. This occurred in around 70% of the units that responded (Figure 3). 

## 4. Discussion

Our survey showed that the majority of preterm infants in Australian and New Zealand neonatal units were supplemented with around 400 IU/day of vitamin D, although there were wide variations in individual unit supplementation criteria. Supplementations with modest doses of vitamin D (maximum intake up to 400 IU/day) have previously been shown to achieve adequate bone mineralisation, and similar serum vitamin D concentrations in preterm infants when compared with higher doses of 800–1000 IU/day [13,14,15]. In an Australian observational study of moderately preterm infants, total intakes of 600 IU/day resulted in lower proportions of vitamin D-deficient infants at 36 weeks PMA than at birth [16]. In more recent randomized controlled trials, Natarajan et al. [17] suggested that 800 IU/day reduced the incidences of vitamin D deficiencies at term CA but showed no improvements in bone mineralization at term and three months CA. Anderson-Berry et al. [18] showed greater improvements in serum vitamin D concentrations and bone density measurements of infants born <32 weeks supplemented with 800 IU, compared to 400 IU/day.

Data are lacking on Vitamin D supplementations in extremely low birth weight (ELBW, <1000 g) infants. Fort et al. found that although greater prevention of vitamin D deficiencies in ELBW infants was achieved at day 14 by supplementations of 800 IU/day, higher than desired concentrations of serum vitamin D were seen at 28 days of age [19]. The authors suggested that higher initial doses for shorter durations to restore vitamin D concentrations to normal, followed by lower dosages, may be an ideal regime for ELBW infants.

The majority of units supplemented iron in preterm infants in line with current recommendations for the first 12 months, although, in some units, iron intakes were double the recommendations at the time of discharge. A 2012 Cochrane review did not find any evidence that an iron dose greater than 3 mg/kg/day resulted in improved haematological outcomes, and such doses may cause potential oxidative damage due to poor regulation of iron absorptions in ELBW infants in the first month of life [10].

The optimal dose, timing, and duration of iron supplementation is yet to be elucidated, and large-scale randomized trials are required. The exact dosages and timings of initiation may vary depending on birth weights and gestational ages, with extremely preterm or growth-restricted infants having higher requirements due to greater post-natal growth velocities. Most units commenced prophylactic enteral iron supplementations (either via human milk fortifiers containing iron or as ferrous sulphate) within the first month after birth, coinciding with the achievement of full, fortified enteral feeds. Early supplementations (within 2–3 weeks of age) have been associated with smaller drops in haemoglobin and ferritin and lowered frequencies of blood transfusions, compared with late supplementations (greater than four weeks) [20]. A five-year follow-up study of VLBW infants randomized to early or late iron supplementations showed trends toward beneficial effects on long-term neurocognitive and psychomotor development with early supplementations [21]. 

Haematological benefits of early supplementations have also been shown in LBW infants [22,23], and follow-up studies have suggested long-term benefits of behavioral functions at seven years in LBW infants supplemented with 2 mg/kg/day iron from six weeks to six months of age [24]. Currently, there are no recommended nutrient intakes for LBW infants, and our study observed that at least 20% of units did not supplement iron in infants with birth weights greater than 2000 g and/or born moderate-to-late preterm.

Most units did not routinely prescribe other additional vitamins or minerals, likely due to nutritional products meeting current nutritional recommendations and a lack of level 1 evidence for further supplementations in preterm infants. Four units (15%) prescribed enteral vitamin E, in addition to fortified feeds. A 2003 Cochrane database review did not support the routine use of high-dose vitamin E given intravenously due to increased late-onset sepsis [25]. No large, randomized controlled trials on enteral vitamin E supplementations and clinical outcomes have been conducted.

Additional calcium and phosphate were routinely supplemented in four units (15%), and additional folic acid in three units (11%), in our survey. A survey of enteral nutrition practices in Australasian neonatal units conducted in 2008 showed that 13% of units routinely supplemented phosphate and 29% prescribed folic acid [26]. This was despite there being sufficient folate in breast milk fortifiers and preterm formulas. We found no evidence in the literature suggesting supplementations of these additional micronutrients at levels higher than estimated requirements were beneficial.

The incidental supplementations of vitamin A in many units using vitamin D-containing multivitamins resulted in vitamin A intakes slightly above current recommended levels but well below levels considered to be toxic. A meta-analysis of relatively high-dose vitamin A supplementations in VLBW infants concluded that the incidence of oxygen requirements by 36 weeks PMA was reduced, and there was a trend towards reductions of ROP and sepsis [27]. The absorption of vitamin A by the preterm gut is known to be poor; therefore, most studies have used intramuscular injections of vitamin A. However, the intramuscular route of administration is not widely accepted because of the discomfort associated with repeated injections. An RCT is currently underway to determine the effect of an enteral water-soluble vitamin A on BPD [28].

One strength of this survey was the high response rate from dietitians working in NICUs; however, there were lower response rates from SCNs. A number of dietitians working in smaller units were not aware of unit guidelines or criteria for vitamin or mineral supplementations. Another limitation of this survey was that information on doses of supplements such as calcium, phosphate, folate, and vitamin E was not collected.

## 5. Conclusions

Whilst supplementations of vitamin D and iron are universal, there is significant variation in practice between neonatal units. This may contribute to intakes of certain micronutrients being above or below recommended ranges for gestational ages or birth weights. The variations in practice may arise from differences in recommended vitamin and mineral intakes between expert groups and a lack of evidence supporting recommendations for supplementations. Whilst recent research suggests that pharmacological doses of certain vitamins may improve clinical outcomes, further research is required to determine optimal doses and timing of deliveries. Such evidence is needed to standardize nutrition practices, contribute to the formulation of micronutrient supplements tailored to preterm infants, and, therefore, prevent under or over-supplementations in this vulnerable population. 

## Figures and Tables

**Table 1:** Recommended vitamin and mineral intakes for fully enterally-fed preterm very low birth weight (VLBW) infants.

Nutrient	Koletzko 2014 [1] (Per kg/Day)	ESPGHAN 2010 [2] (Per kg/Day)	Tsang 2005 [3] (Per kg/Day)
Vitamin A (IU)	1332–3663	1320–3300	700–1500
Vitamin E (mg α-TE)	2.2–11	2.2–11	4–8
Vitamin D (IU)	400–1000 per day from milk and supplement	800–1000 per day	150–400
Folic acid (µg)	35–100	35–100	25–50
Iron (mg)	2–3	2–3	2–4
Zinc (mg)	1.4–2.5	1.1–2.0	1–3
Calcium (mg)	120–200	120–140	100–220
Phosphorus (mg)	60–140	60–90	60–140

IU, International Units; α-TE, alpha-tocopherol equivalents; ESPGHAN, European Society for Paediatric Gastroenterology Heptatology and Nutrition.

**Table 2:** Ferrous sulphate doses prescribed for inpatients and at discharge.

Dose (mg/kg/day)	Inpatients *N* = 29	At Discharge *N* = 29
1.8	4 (14%)	5 (17%)
2.4	3 (10%)	3 (10%)
3	17 (59%)	17 (59%)
6	2 (7%)	3 (10%)
Not specified	3 (10%)	1 (3%)

## Supplementary Materials

The following are available online at https://www.mdpi.com/2072-6643/12/1/51/s1, Supplementary material: Neonatal Dietitians Part 2 Survey Questions.
