# Supplementary material for: Vitamin and Mineral Supplementation Practices in Preterm Infants: A Survey of Australian and New Zealand Neonatal Intensive and Special Care Units
Source: Nutrients. 2019 Dec 23;12(1):51. doi: 10.3390/nu12010051 (PMC7019934; doi:10.3390/nu12010051)
Supplement: Supplementary file 1 [file nutrients-12-00051-s001.pdf]

Table 1 Survey questions

Part 2

**1.**	**Does your unit have a written policy on the following:**	
	Screening criteria for dietitian referral	Yes/No
	Enteral Feeding	Yes/No
	Intravenous feeding	Yes/No
	Withholding feeds	Yes/No
	Gastric aspirate volumes	Yes/No
	Vitamin and mineral supplementation (inpatient)	Yes/No
	Other	please specify
**2.**	**Which country is your hospital in?**	
	New Zealand	
	Australia	
**3.**	**What is the name of the hospital where you work?**	
**4.**	**In your unit, is a vitamin D supplement prescribed for preterm infants? If yes, what is the criteria (birthweight, gestational age, other)?**	
**5.**	**What is the name of the inpatient vitamin D supplement and dose (in ml)**	
	Vitadol C	
	Pentavite	
	Puria	
	Other (please specify)	
**6.**	**Is vitamin D prescribed on discharge? If yes, what is the criteria for who gets it?**	
**7.**	**If yes, what is the name of the vitamin D supplement and dose (in ml) on discharge?**	
	Vitadol C	
	Pentavite	
	Puria	
	Other (please specify)	
**8.**	**When is this Vitamin D stopped? (ie for how long is the vitamin D supplement recommended?)**	
**9.**	**In your unit, is ferrous sulphate prescribed for preterm infants? If yes, what is the criteria (birthweight, gestational age, other?)**	
**10.**	**What is the inpatient prophylactic (baby not anaemic) ferrous sulphate dose (in ml)**	
**11.**	**Is ferrous sulphate prescribed on discharge? If yes, what is the criteria for who gets it?**	
**12.**	**If yes, what is the prophylactic (baby not anaemic) ferrous sulphate dose (in ml) on discharge?**	
**13.**	**When is this ferrous sulphate stopped? (ie for how long is the ferrous sulphate supplement recommended?)**	
**14.**	**What other vitamin and mineral supplements are routinely prescribed (without proven deficiency) in your neonatal unit?**<br>Folic acid<br>Calcium<br>Phosphate<br>Zinc<br>Vitamin E<br>Vitamin A (not as Vitadol C or Pentavite)<br>Other (please specify)	
